# Enantiopure Indolo[2,3-*a*]quinolizidines: Synthesis and Evaluation as NMDA Receptor Antagonists

**DOI:** 10.3390/molecules21081027

**Published:** 2016-08-06

**Authors:** Nuno A. L. Pereira, Francesc X. Sureda, Maria Pérez, Mercedes Amat, Maria M. M. Santos

**Affiliations:** 1 Instituto de Investigação do Medicamento (iMed.ULisboa), Faculdade de Farmácia, Universidade de Lisboa, Av. Prof. Gama Pinto, 1649-003 Lisboa, Portugal; nalpereira@ff.ulisboa.pt; 2 Pharmacology Unit, Faculty of Medicine and Health Sciences, Universitat Rovira i Virgili, C./St. Llorenç 21, 43201 Reus (Tarragona), Spain; francesc.sureda@urv.cat; 3 Laboratory of Organic Chemistry, Faculty of Pharmacy and Institute of Biomedicine (IBUB), University of Barcelona, Av. Joan XXIII, s/n, 08028 Barcelona, Spain; mariaperez@ub.edu (M.P.); amat@ub.edu (M.A.)

**Keywords:** indoloquinolizidines, 1,2-aminoalcohols, tryptophanol, NMDA receptor, antagonists

## Abstract

Enantiopure tryptophanol is easily obtained from the reduction of its parent natural amino acid trypthophan (available from the chiral pool), and can be used as chiral auxiliary/inductor to control the stereochemical course of a diastereoselective reaction. Furthermore, enantiopure tryptophanol is useful for the syntheses of natural products or biological active molecules containing the aminoalcohol functionality. In this communication, we report the development of a small library of indolo[2,3-*a*]quinolizidines and evaluation of their activity as *N*-Methyl d-Aspartate (NMDA) receptor antagonists. The indolo[2,3-*a*]quinolizidine scaffold was obtained using the following key steps: (i) a stereoselective cyclocondensation of (*S*)- or (*R*)-tryptophanol with appropriate racemic δ-oxoesters; (ii) a stereocontrolled cyclization on the indole nucleus. The synthesized enantiopure indolo[2,3-*a*]quinolizidines were evaluated as NMDA receptor antagonists and one compound was identified to be 2.9-fold more potent as NMDA receptor blocker than amantadine (used in the clinic for Parkinson’s disease). This compound represents a hit compound for the development of novel NMDA receptor antagonists with potential applications in neurodegenerative disorders associated with overactivation of NMDA receptors.

## 1. Introduction

Chiral pool synthesis uses chiral natural products by incorporating part of them into the target structure. As both enantiomers of the desired final product can be potentially generated, compounds from the chiral pool are extremely valuable and versatile in asymmetric synthesis.

**Scheme 1:**
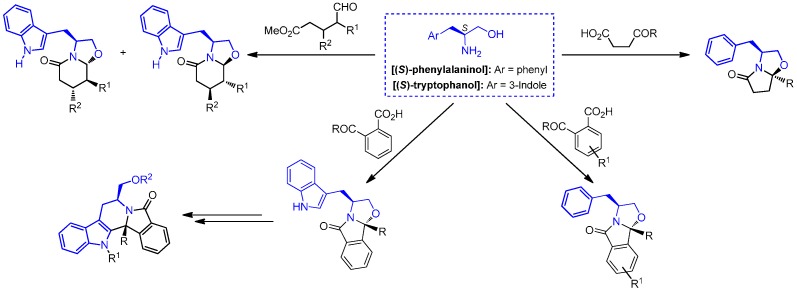
Synthesis of biological active small molecules starting from enantiopure 1,2-aminoalcohols (tryptophanol and phenylalaninol).

A wide range of enantiopure amino acids, isolated from natural sources, have been used in academia and pharmaceutical companies as chiral auxiliaries/inductors to synthesize biologically active enantiopure compounds [1]. The asymmetric syntheses of natural products, or biological active molecules can also be achieved using enantiopure 1,2-aminoalcohols containing a stereogenic centre (which can be obtained by reduction of the parent natural amino acids) as chiral inductors [2]. In the last few years we have developed several novel bioactive compounds in this area of research starting from the enantiopure 1,2-aminoalcohols tryptophanol and phenylalaninol. Using this versatile synthetic approach we have developed libraries of enantiopure phenylalaninol-derived oxazolopyrrolidone lactams [3], tryptophanol-derived oxazolopiperidone lactams [4,5], oxazoloisoindolinones [6,7], and indolizinoindolones [8] designed to act on important therapeutic targets (Scheme 1).

**Figure 1:**
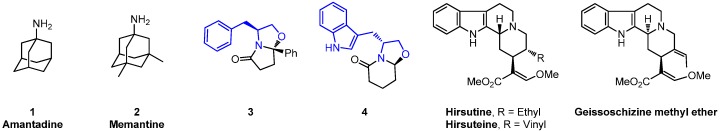
Chemical structure of *N*-methyl-d-aspartate receptor antagonists and natural products hirsutine, hirsuteine, and geissoschizine methyl ether.

One area of research that we are particularly interested is in the development of small molecules that control exacerbated *N*-methyl-d-aspartate (NMDA) receptor activity. This area of research is incredibly challenging, as these ligand-gated ion channels exhibit a complex pharmacology and molecular architecture, which renders most antagonists unsuitable for therapeutic use [9]. Besides playing a crucial role in the biochemical cascade signaling behind the development of neurodegenerative disorders such as Alzheimer’s and Parkison’s diseases, NMDA receptors are also extremely important in sustaining healthy memory, learning and cognition processes [10]. Therefore, compounds that can suppress NMDA receptor activity during glutamate-induced excitotoxicity episodes, but leave its normal physiological role unharmed, are of great interest [11]. These constraints are quite difficult to overcome and to date only a handful of clinically tolerated molecules exist, such as amantadine (**1**) and memantine (**2**) (Figure 1) [12,13].

In the last few years, starting from chiral 1,2-aminoalcohols, we have developed libraries of novel enantiopure bicyclic lactams which were screened for activity as NMDA receptor antagonists. From our previous screenings, two hit compounds (**3** and **4**) were identified to block NMDA receptor activity, presenting IC_50_ values of around 60 μM (Figure 1) [3,4].

**Figure 2:**
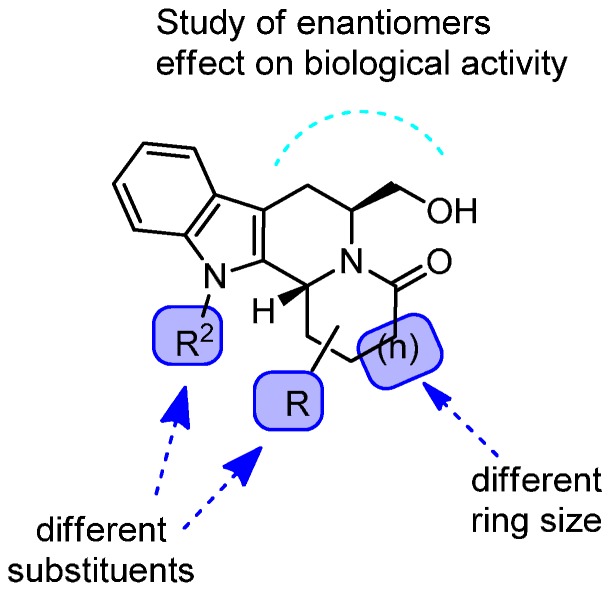
Compounds to be synthesized.

Following this work, we were interested in studying the indolo[2,3-*a*]quinolizidine scaffold obtained by cyclization of tryptophanol-derived oxazolopiperidones (Figure 2). This interest resulted from reports that several indolo[2,3-*a*]quinolizidine natural products protect neurons from NMDAR-mediated death. In fact, some alternatives for the treatment of mental dementia-associated symptoms come from long-used traditional medicines such as extracts from *Uncaria* plant species. These were found to contain biologically active compounds protecting neurons from NMDAR-mediated death [14]. In particular, the indolo[2,3-*a*]quinolizidines hirsutine and hirsuteine (Figure 1), present in *choto-san* and *yokukansan* extracts, reduce NMDAR-mediated Ca^2+^ neural overload [15,16]. Another indolo[2,3-*a*]quinolizidine present in these plant extracts is geissoschizine methyl ether, which besides presenting neuroprotective activity similar to that observed with hirsutine and hirsuteine is able to cross the blood-brain barrier by oral administration [17]. Although some of the fundamental pharmacological mechanisms through which these natural products exert their biological activity have been demonstrated [18], to the best of our knowledge, no studies have been performed with the indolo[2,3-*a*]quinolizidine nucleus reported herein. In this communication, we present the synthesis of a series of enantiopure indolo[2,3-*a*]quinolizidine derivatives, and their evaluation as NMDA receptor antagonists. In order to perform an initial structure-activity relationship (SAR) study, particularly directed to understand the structural features for optimal inhibition of NMDA receptor activity, we synthesized: (i) compounds containing different substituents at the piperidinone ring; (ii) pairs of enantiomers; (iii) compounds with different ring size of the lactam; (iv) compounds with the indole nitrogen free and protected (Figure 2).

## 2. Results and Discussion

### 2.1. Chemistry

The asymmetric synthesis of indoloquinolizidines was achieved starting from enantiopure tryptophanol, a methodology used previously by the groups of Allin [19,20] and Amat-Bosch [21,22,23,24,25,26,27] for the synthesis of several indole alkaloids. In this synthetic strategy, tryptophanol is used not only as the source of chirality, but is also used to incorporate the tryptamine moiety present in the target alkaloids [28,29].

**Scheme 2:**
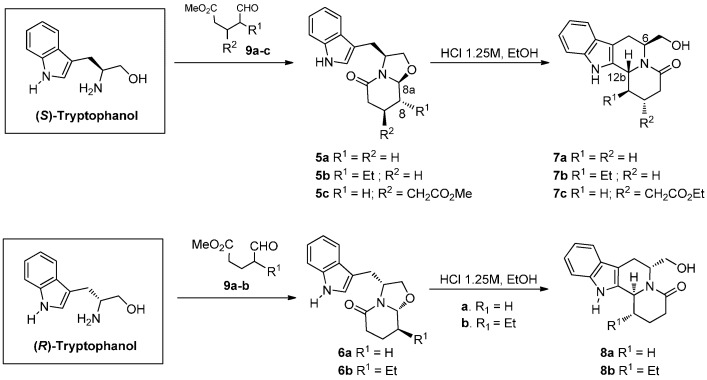
Synthesis of indolo[2,3-*a*]quinolizidines **7a**–**c**, and **8a**–**b**.

In order to explore the potential of the indolo[2,3-*a*]quinolizidine scaffold for the development of novel NMDA receptor antagonists, we synthesized a series of compounds containing different substituents (H, Et and CH_2_CO_2_Et) around the indolo[2,3-*a*]quinolizidine skeleton, starting from enantiopure tryptophanol (Scheme 2).

Reaction of (*S*)-tryptophanol with δ-oxoacid derivatives **9a**–**c** afforded bicyclic lactams **5a**, **5b** and **5c**, along with minor amounts of the respective diastereoisomers at the 8a position, 8,8a positions and 7,8a positions, respectively. The relative stereochemistry between H-3 and H-8a in the major lactams **5a**, **5b**, and **5c** is *cis* (Scheme 2) [19,22,23]. Stereocontrolled cyclization of bicyclic lactam **5a** with 1.25M HCl, led to the formation of indolo[2,3-*a*]quinolizidine **7a** as a single diastereoisomer (Scheme 2) [19,20]. Cyclization of bicyclic lactam **5b** by intramolecular α-amidoalkylation on the indole 2-position using HCl led to 6,12b-*trans* indoloquinolizidine **7b** [22]. Not surprisingly, the cyclization of bicyclic lactam **5c** in the presence of HCl-EtOH caused transesterification of the ester moiety and led to indoloquinolizidine **7c** with very good yield (97%).

Bearing in mind the importance of the absolute stereochemistry of biologically active compounds, we have also prepared enantiomers **8a**–**b** starting from enantiopure (*R*)-tryptophanol-derived oxazolopiperidones **6a**–**b**. Reaction of (*R*)-tryptophanol with δ-oxoacid derivative **9a** afforded bicyclic lactam **6a** and the 8,8a-diastereoisomer **6a’** in 60% yield as a 5:1 mixture of separable diastereoisomers. Similar results were obtained with racemic oxoester **9b** affording enantiopure lactam **6b** in 58% yield, along with minor amounts of the 8,8a-diastereoisomer **6b’** (12%), in a process involving a dynamic kinetic resolution with epimerization of the stereogenic center α to the aldehyde carbonyl group (Scheme 2) [4]. Cyclization of bicyclic lactams **6a** and **6b** with 1.25M HCl, led to the formation of indolo[2,3-*a*]quinolizidines **8a** and **8b**, respectively.

**Scheme 3:**
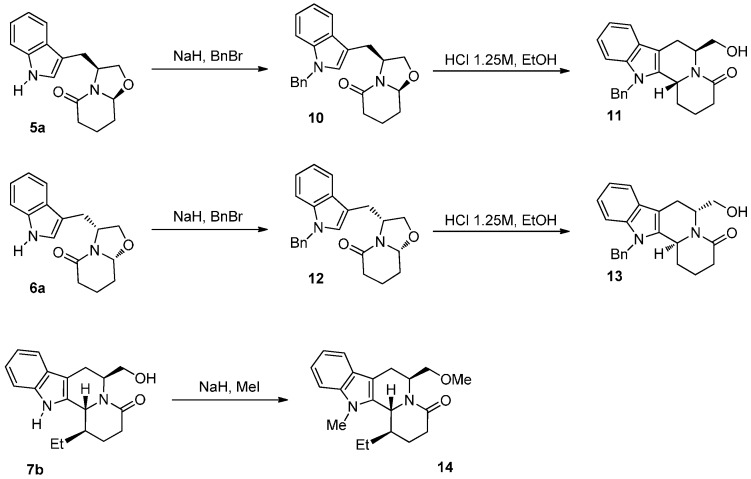
Synthesis of compounds **11**, **13**, and **14**.

We then decided to synthesize compounds **11** and **13**, *N*-indole protected derivatives of indolo[2,3-*a*]quinolizidines **7a** and **8a**, respectively, to better understand the structural requisites for NMDA receptor antagonistic activity. Compound **11** and the enantiomer **13** were obtained in 80% yield via intramolecular amidoalkylation of intermediates **10** and **12**, respectively (Scheme 3). Taking advantage of the N-H and O-H polarized bonds present in the scaffold of our target molecules, compound **14** was easily synthesized, by reaction of indoloquinolizidine **7b** with methylene iodide, with 74% yield.

**Scheme 4:**
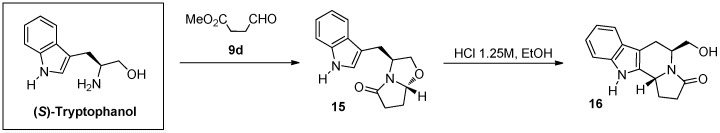
Synthesis of compound **16**.

Moreover, in order to explore the fused piperidone ring contraction effect on NMDA receptor antagonism activity, we synthesized compound **16** containing a fused pyrrolidone ring by intramolecular amidoalkylation of bicyclic lactam **15** (obtained by reaction of (*S*)-tryptophanol with methyl 4-oxobutanoate **9d**, Scheme 4).

### 2.2. Biological Activity

**Figure 3:**
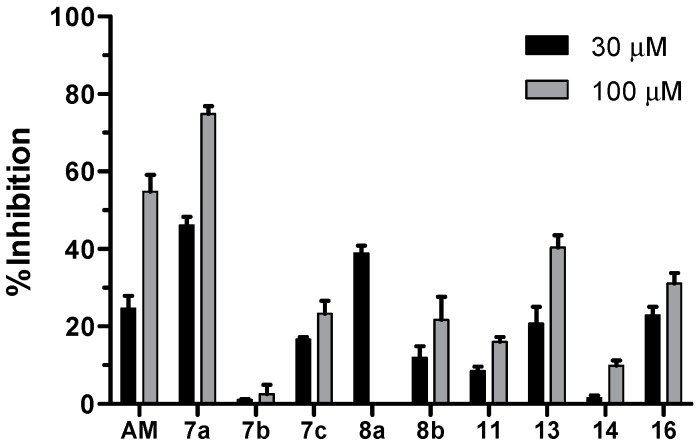
Inhibitory effect of compounds **7a**–**c**, **8a**–**b**, **11**, **13**, **14**, **16** on *N*-Methyl d-Aspartate (NMDA)-induced intracellular calcium increase in cultured cerebellar granule neurons are shown at 30 μM (black) and 100 μM (dark grey) of each compound. Amantadine (AM) at 30 μM and 100 μM was included in the assay as a positive control. Compound **8a** was not evaluated at 100 μM due to solubility issues.

The NMDA receptor blocking activity of compounds 7**a**–**c**, **8a**–**b**, **11**, **13**, **14** and **16** was evaluated by measuring the ability of the compounds to inhibit the intracellular calcium increase, induced by NMDA, in in vitro cultures of cerebellar granule neurons. Addition of NMDA (100 μM) in the presence of glycine (10 μM) produced a robust and stable increase in intracellular calcium, which was challenged with the compounds to be tested (Figure 3). Compounds **7a** and **8a**, without any substituent in the piperidone ring, were more active than amantadine. By contrast, compounds with an ethyl group at C-1 (compounds **7b** and **8b**) or a CH_2_CO_2_Et at C-2 (compound **7c**) inhibited less than 30% the NMDA-induced intracellular calcium increase. Except for compound **7a**, the compounds derived from (*R*)-tryptophanol were more active than the corresponding enantiomers, derived from (*S*)-tryptophanol (**7b** and **11** versus **8b** and **13**, respectively). Compounds **11** and **13**, which have the *N*-indole protected with a benzyl group, were less active than the corresponding unprotected indoloquinolizidines **7a** and **8a**, respectively, suggesting that the indole N-H can be important for the NMDA receptor antagonist activity or that the benzyl group is too bulky for the binding pocket in the NMDA receptor. The same result was observed for compound **14**, which was less active than the unprotected indoloquinolizidine **7b**. Compound **16,** with a pyrrolidone ring, lost activity compared with the piperidone **7a** counterpart, which further supports the importance of the piperidone ring for NMDA receptor inhibitory activity, previously observed with tryptophanol-derived oxazolopiperidone lactams [4].

**Figure 4:**
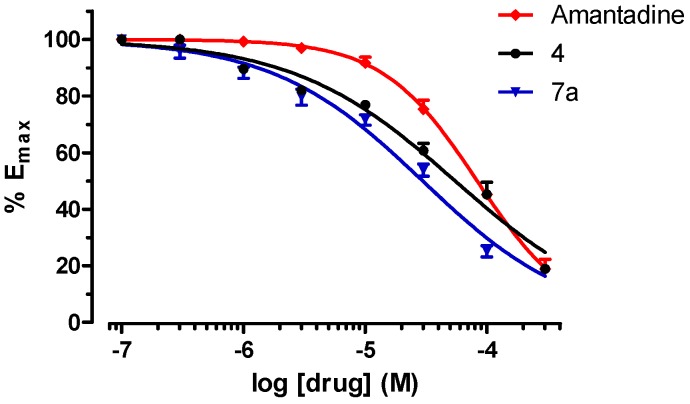
Inhibitory effect on NMDA-induced intracellular calcium increase in cultured cerebellar granule neurons treated with increasing doses of amantadine, **4** and **7a**.

The IC_50_ value was determined for the most active compound (compound **7a**, Figure 4). The IC_50_ value obtained for indoloquinolizidine was 30.4 μM (Table 1), representing a 2.9-fold increase of activity compared with amantadine (IC_50_ = 88.5 μM) and a 2-fold increase of activity compared with tryptophanol-derived lactam **4** (IC_50_ = 63.4 μM).

## 3. Materials and Methods

### 3.1. General Information

(*L*)-tryptophanol [(*S*)-tryptophanol] was bought from Sigma-Aldrich (Schnelldorf, Bavaria, Germany). Bicyclic lactams **5a**–**c** [19,22] and δ-oxo-esters **9a**–**c** [30,31] were synthesized as described in the literature. Methyl 4-oxobutanoate (**9d**) was synthesized using the method described for the synthesis of methyl 5-oxopentanoate (**9a**) but starting from γ-butyrolactone. Evaporation of solvents was accomplished with a rotatory evaporator. Thin-layer chromatography was done on SiO_2_ (silica gel 60 F254), and the spots were located by UV. For column chromatography silica gel 200–400 mesh was used. ^1^H- and ^13^C-NMR spectra were recorded on a Bruker 400 MHz Ultra-Shield (Wissembourg, Bas-Rhin, France). ^1^H- and ^13^C-NMR chemical shifts are reported as δ values, in parts per million (ppm) referenced to the solvent used. Data are reported in the following manner: chemical shift, multiplicity, coupling constant (J) in hertz (Hz), integrated intensity, and assignment (when possible). Multiplicities are reported using the following abbreviations: s, singlet; d, doublet; t, triplet; q, quartet; dd, doublet of doublets; tt, triplet of triplets; m, multiplet. Spectra were assigned using appropriate COSY, DEPT and HMQC sequences. Microanalysis were performed in a Thermo ScientificTM FLASH 2000 Series CHNS/O analyser (Waltham, MA, USA) and are within ±0.5% of theoretical values.

### 3.2. Synthesis of (R)-Tryptophanol

LiAlH_4_ (5 g, 134.64 mmol) was slowly added to a suspension of (d)-tryptophan (5 g, 24.48 mmol) in THF (200 mL) at 0 *°*C. After 30 min, the mixture was heated at reflux overnight. The resulting mixture was cooled to 0 *°*C and a saturated aqueous solution of Na_2_SO_4_ was added. The suspension was filtered, and the filtrate extracted with EtOAc (250 mL). The aqueous phase was washed with EtOAc (3 × 100 mL), and the combined organic phases were dried, and concentrated to give (*R*)-tryptophanol (4.58 g, 98%).

### 3.3. General Procedure for the Synthesis of Compounds ***6a**–**b***, and ***15***

A solution of (*R*)- or (*S*)-tryptophanol (1 equiv), and γ-oxo-ester (1.1 equiv) in toluene was heated at reflux, under Dean-Stark conditions, till consumption of the tryptophanol. The solvent was removed under reduced pressure and the residue obtained was purified by flash chromatography on silica gel [4,22].

*[(3R,8aR)-3-((1H-Indol-3-yl)methyl)tetrahydro-2H-oxazolo[3,2-a]pyridin-5(3H)-one)* (**6a**). Following the general procedure, starting from (*R*)-tryptophanol (1.17 g, 6.15 mmol), toluene (40 mL) and methyl 5-oxopentanoate (**9a**) (0.88 g, 6.77 mmol). Eluent for flash chromatography: EtOAc/*n*-Hex (1:1). Compound **6a** (0.63 g, 76%): ${\left[\alpha \right]}_{D}^{20}$ = +35.6° (*c* = 1.9, CH_2_Cl_2_); ^1^H-NMR spectra was found to be identical to that obtained for compound **5a** [4,19].

*[(3R,8S,8aR)]-8-Ethyl-3-(3-indolylmethyl)-5-oxo-2,3,6,7,8,8a-hexahydro-5H-oxazolo[3,2-a]pyridine* (**6b**). Following the general procedure, starting from (*R*)-tryptophanol (0.54 g, 2.84 mmol), toluene (20 mL) and methyl 4-formylhexanoate (**9b**) (0.49 g, 3.12 mmol). Eluent for flash chromatography: EtOAc/*n*-Hex (2:1). Compound **6b** (0.54 g, 58%): ${\left[\alpha \right]}_{D}^{20}$ = −18.2*°* (*c* = 2.2, CH_2_Cl_2_); ^1^H-NMR spectra was found to be identical to that obtained for compound **5b** [4,22].

*(3S,7aR)-3-((1H-Indol-3-yl)methyl)tetrahydropyrrolo[2,1-b]oxazol-5(6H)-one* (**15**). Following the general procedure, starting from (*S*)-tryptophanol (1 g, 5.26 mmol), toluene (80 mL) and methyl 4-oxobutanoate (**9d**) (0.67 g, 5.78 mmol). Eluent for flash chromatography: EtOAc/*n*-Hex (1:1). Compound **15** was obtained after recrystallization in EtOAc (0.45 g, 33%): ${\left[\alpha \right]}_{D}^{20}$ = +67.8*°* (*c* = 1.6, CH_2_Cl_2_); ^1^H-NMR was found to be identical to that obtained for the enantiomer previously described [4]; Anal. calcd. for C_15_H_16_N_2_O_2_: C 70.29, H 6.31, N 10.93, found: C 70.28, H 6.35, N 10.94.

### 3.4. General Procedure for the Synthesis of Compounds ***10*** and ***12***

To a solution of the starting lactam (0.26 mmol, 1.0 equiv.) in anhydrous DMF (3 mL) was added NaH 60% (0.39 mmol, 1.5 equiv.). The mixture was allowed to stir for 15 min and then benzyl bromide (0.05 mL, 1.5 equiv.) was added dropwise. The mixture was stirred at room temperature for 1 h. Water was added, followed by extraction with EtOAc. The combined organic extracts were washed with brine, dried, and concentrated. The crude residue was purified by flash chromatography with the eluent EtOAc/n-hexane (2:1) to give the product as a white solid.

*(3S,8aS)-3-[(1-Benzyl-1H-indol-3-yl)methyl]tetrahydro-2H-oxazolo[3,2-a]pyridin-5(3H)-one* (**10**). Following the general procedure, starting from lactam **5a** (0.07 g, 0.26 mmol). **10** (0.08 g, 86%): ^1^H NMR (CDCl_3_) δ 7.69 (d, *J* = 7.8 Hz, 1H, ar), 7.29 (dd, *J* = 13.8, 6.5 Hz, 5H, ar), 7.18 (t, *J* = 7.2 Hz, 1H, ar), 7.11 (dd, *J* = 13.3, 6.9 Hz, 2H, ar), 6.93 (s, 1H, H-2-indole), 5.29 (m, 2H, N-CH_2_), 4.61 (qd, *J* = 7.8, 3.2 Hz, 1H, H-3), 4.42 (dd, *J* = 8.9, 4.4 Hz, 1H, H-8a), 4.06 (m, 1H, H-2), 3.68 (m, 1H, H-2), 3.30 (dd, *J* = 14.3, 3.2 Hz, 1H, CH_2_-indole), 3.06 (dd, *J* = 14.3, 8.5 Hz, 1H, CH_2_-indole), 2.49 (m, 1H, H-6), 2.30 (m, 1H, H-alkyl, H-6), 2.14 (m, 1H, H-alkyl), 1.84 (m, 1H, H-alkyl), 1.43 (m, 2H, H-alkyl).

*(3R,8aR)-3-[(1-Benzyl-1H-indol-3-yl)methyl]tetrahydro-2H-oxazolo[3,2-a]pyridin-5(3H)-one* (**12**). Following the general procedure, starting from lactam **6a** (0.09 g, 0.33 mmol). **12** (0.11 g, 92%): ^1^H NMR spectra was found to be identical to that obtained for compound **10**.

### 3.5. General Procedure for the Synthesis of Compounds ***7a**–**c***, ***8a**–**b***, ***11***, ***13*** and ***16***

In EtOH, 1.25 M HCl was added to the proper starting lactam and the reaction mixture was stirred at room temperature till consumption of the starting material. The solvent was evaporated and the resulting mixture was dissolved in EtOAc and washed with saturated aqueous NaHCO_3_. After extraction with EtOAc, the combined organic extracts were washed with H_2_O, dried, and concentrated to give a precipitate. The precipitate was washed with cold EtOAc and recrystallized from the adequate solvent.

*(6S,12bR)-6-(Hydroxymethyl)-1,2,3,6,7,12b-hexahydroindolo[2,3-a]quinolizin-4(12H)-one* (**7a**). Following the general procedure, starting from lactam **5a** (0.18 g, 0.66 mmol) and 1.25 M HCl in EtOH (2.6 mL). Reaction time: 24 h. Recrystallized from EtOAc/*n*-hexane to yield a yellow solid **7a** (0.146 g, 80%): ${\left[\alpha \right]}_{D}^{20}$ = +143.6° (*c* = 2.1, MeOH); ^1^H-NMR spectra was identical to that described previously [20]; Anal. calcd. C_16_H_18_N_2_O_2_: C 71.08, H 6.73, N 10.37, found: C 70.91, H 6.81, N 10.26.

*(1R,6S,12bR)-1-Ethyl-6-(hydroxymethyl)-1,2,3,6,7,12b-hexahydroindolo[2,3-a]quinolizin-4(12H)-one* (**7b**). Following the general procedure, starting from lactam **5b** (0.2 g, 0.67 mmol) and 1.25 M HCl in EtOH (2.7 mL). Reaction time: 24 h. Recrystallized from EtOH as a white solid **7b** (0.138g, 69%): ^1^H-NMR was found to be identical to that described previously [22]; Anal. Calcd for C_18_H_22_N_2_O_2_: C, 72.46; H, 7.43; N, 9.39. Found: C, 72.19; H, 7.39; N, 9.24.

*Ethyl 2-((2R,6S,12bR)-6-(hydroxymethyl)-4-oxo-1,2,3,4,6,7,12,12b-octahydroindolo[2,3-a]-quinolizin-2-yl)acetate* (**7c**). Following the general procedure and starting from lactam **5c** (0.05 g, 0.15 mmol) and 1.25 M HCl in EtOH (3.5 mL). Reaction time: 24 h. Recrystallized from CHCl_3_ as a white solid (0.047 g, 97%); mp 109.5 °C–112 °C; IR (KBr) 3256 (NH), 1730 (C=O, acid), 1618 (C=O, amide) cm^−1^; ^1^H-NMR (400 MHz, CDCl_3_ with a drop of CD_3_OD) δ 7.42 (d, *J* = 7.8 Hz, 1H, H-ar), 7.29 (d, *J* = 8.0 Hz, 1H, H-ar), 7.13 (t, *J* = 7.5 Hz, 1H, H-ar), 7.05 (t, *J* = 7.4 Hz, 1H, H-ar), 5.43–5.32 (m, 1H, H-6), 4.73 (d, *J* = 10.6 Hz, 1H, H-12b), 4.14 (q, *J* = 7.1 Hz, 2H, CH_2_CH_3_), 3.64–3.49 (m, 2H, OCH_2_), 2.92 (dd, *J* = 15.9, 4.9 Hz, 1H, H-7), 2.68 (m, 2H, H-7 & H-alkyl), 2.60 (d, *J* = 12.6 Hz, 1H, H-alkyl), 2.45 (m, 1H, H-2), 2.31 (m, 2H, CH_2_CO_2_Et), 2.11 (dd, *J* = 17.3, 12.2 Hz, 1H, H-alkyl), 1.43 (m, 1H, H-alkyl), 1.26 (t, *J* = 7.1 Hz, 3H, CH_2_CH_3_); ^13^C-NMR (100 MHz, CDCl_3_) δ 172.16 (C=O), 170.42 (C=O), 136.52 (C-q), 131.47 (C-q), 126.79 (C-q), 121.92 (CH-ar), 119.36 (CH-ar), 118.10 (CH-ar), 111.03 (CH-ar), 105.88 (C-q), 61.55 (OCH_2_), 60.87 (CH_2_CH_3_), 50.20 (C-12b), 49.04 (C-6), 39.97 (CH_2_CO_2_CH_2_CH_3_), 38.38 (CH_2_-alkyl), 34.72 (CH_2_-alkyl), 28.17 (C-2), 21.30 (C-7), 14.11 (CH_2_CH_3_); Anal. calcd. for: C_20_H_24_N_2_O_4_·0.75H_2_O: C 64.92, H 6.69, N 7.57, found: C 64.62, H 6.73, N 7.41.

*(6R,12bS)-6-(Hydroxymethyl)-1,2,3,6,7,12b-hexahydroindolo[2,3-a]quinolizin-4(12H)-one* (**8a**). Following the general procedure, and starting from lactam **6a** (0.1 g, 0.37 mmol) and 1.25 M HCl in EtOH (3.5 mL). Reaction time: 24 h. Recrystallized from EtOAc/*n*-hexane as a white solid **8a** (0.075 g, 75%): ${\left[\alpha \right]}_{D}^{20}$ = −143.3° (c = 1.9, MeOH); ^1^H-NMR spectra was found to be identical to that obtained for compound **7a**; Anal. Calcd. C_16_H_18_N_2_O_2_·0.25H_2_O: C 69.92, H 6.80, N 10.20, found: C 69.80, H 6.62, N 10.01.

*(1S,6R,12bS)-1-Ethyl-6-(hydroxymethyl)-1,2,3,6,7,12b-hexahydroindolo[2,3-a]quinolizin-4(12H)-one* (**8b**). Following the general procedure, and starting from lactam **6b** (0.05 g, 0.17 mmol) and 1.25 M HCl in EtOH (3.5 mL). Reaction time: 20 h. Recrystallized from EtOAc/*n*-hexane as a white solid **8b** (0.046 g, 92%): The ^1^H-NMR spectra was found to be identical to that to that obtained for compound **7b**.

*(6S,12bR)-12-Benzyl-6-(hydroxymethyl)-1,2,3,6,7,12b-hexahydroindolo[2,3-a]quinolizin-4(12H)-one* (**11**). Following the general procedure, and starting from lactam **10** (0.07 g, 0.19 mmol) and 1.25 M HCl in EtOH (1.3 mL). Reaction time: 20 h. Recrystallized from EtOAc/n-hexane to yield a white solid **11** (0.056 g, 80%): ^1^H-NMR was found to be identical to that described in the literature [30]; Anal. calcd. for: C_23_H_24_N_2_O_2_·0.25H_2_O: C 75.69, H 6.78, N 7.68, found: C 75.65, H 6.50, N 7.71.

*(6R,12bS)-12-Benzyl-6-(hydroxymethyl)-1,2,3,6,7,12b-hexahydroindolo[2,3-a]quinolizin-4(12H)-one* (**13**). Following the general procedure, and starting from lactam **12** (0.11 g, 0.32 mmol) and 1.25 M HCl in EtOH (2 mL). Reaction time: 20 h. Recrystallized from EtOAc/*n*-hexane to yield a white solid **13** (0.092 g, 80%): The ^1^H-NMR spectra was found to be identical to that to that obtained for compound **11**; Anal. calcd. for: C_23_H_24_N_2_O_2_: C 76.64, H 6.71, N 7.77, found: C 76.55, H 6.58, N 7.84.

*(5S,11bR)-5-(Hydroxymethyl)-5,6,11,11b-tetrahydro-1H-indolizino[8,7-b]indol-3(2H)-one* (**16**). Following the general procedure, and starting from lactam **15** (0.14 g, 0.55 mmol) and 1.25 M HCl in EtOH (4 mL). Reaction time: 18 h. Recrystallized from EtOAc to yield a white solid **16** (0.05 g, 36%): ^1^H-NMR was found to be identical to that described in the literature [32]; Anal. calcd. for C_15_H_16_N_2_O_2_·0.15H_2_O: C 69.56, H 6.36, N 10.93, found: C 69.40, H 6.32, N 10.43.

### 3.6. Synthesis of (1R,6S,12bR)-1-Ethyl-6-(methoxymethyl)-12-methyl-1,2,3,6,7,12b-hexahydroindolo [2,3-a]quinolizin-4(12H)-one (***14***)

NaH (3 eq., 60% dispersion in mineral oil) was added to a solution of the indoloquinolizidine **7b** (0.11 g, 0.37 mmol) in DMF (5 mL). After stirring for 30 min, MeI (3 eq., 0.08 mL) was added dropwise whilst maitaining the solution under inert atmosphere and an ice bath. The reaction mixture was allowed to stir at room temperature for 16 h and after this period was quenched with ice cold deionized water (20 mL). The mixture was then extracted three times with EtOAc (3 × 20 mL). The gathered extracts were washed with brine (30 mL) and dried over Na_2_SO_4_ before evaporating the solvent to dryness. The crude compound was then purified by flash chromatography using EtOAc/*n*-Hex (3:1). The precipitate was recrystallized from EtOAc/*n*-Hex. **14** (0.089 g, 74%); mp 141 °C–143 °C; IR (KBr) 1638 (C=O) cm^−1^; ^1^H-NMR (400 MHz, CDCl_3_) δ 7.54 (d, *J* = 8.0 Hz, 1H, ar), 7.32 (d, *J* = 8.0 Hz, 1H, ar), 7.28–7.22 (m, 1H, ar), 7.15 (m, 1H, ar), 5.32 (m, 1H, H-6), 4.54 (d, *J* = 6.6 Hz, 1H, H-12b), 3.70 (s, 3H, NCH_3_), 3.30–3.16 (m, 4H, OCH_3_ and CH_2_OH), 3.03 (dd, *J* = 9.5, 8.2 Hz, 1H, CH_2_OH), 2.94 (d, *J* = 2.8 Hz, 2H), 2.68–2.43 (m, 2H), 2.10–1.89 (m, 2H), 1.72–1.41 (m, 3H), 0.92 (t, *J* = 7.3 Hz, 3H, CH_2_CH_3_); ^13^C-NMR (100 MHz, CDCl_3_) δ 172.03 (C=O), 138.15 (C-q), 133.16 (C-q), 127.03 (C-q), 121.84 (CH-ar), 119.54 (CH-ar), 118.40 (CH-ar), 109.25 (CH-ar), 108.50(C-q), 71.21 (CH_2_OH), 58.70 (OCH_3_), 54.61 (C-12b), 47.64 (C-6), 42.99 (C-1), 31.57 (NCH_3_), 29.71 (CH_2_), 25.42 (CH_2_), 22.91 (CH_2_), 21.61 (CH_2_), 11.97 (CH_2_CH_3_).

### 3.7. NMDA Receptor Antagonist Activity

The activity of the synthesized compounds as NMDA receptor antagonists was evaluated using primary cultures of rat cerebellar neurons. Cultures were prepared from 7–8 day-old Wistar rats (Charles River, Saint-Germain-sur-l’Arbresle, France). Cerebella were dissected, minced and trypsinized, and after several sedimentations, cells were plated on poly-lysinized coverslips placed in 24-well plates at a density of 1 *×* 10^6^ cells/mL. Plates were kept at 37 °C in a cell incubator (Sanyo, Gunma, Japan). After 16–18 h, 10 μM cytosine arabinoside (Sigma-Aldrich, USA) was added to avoid excessive proliferation of astrocytes. Cultures prepared in this manner are ready to be used in the NMDA receptor activity assays from the 6th to the 10th day in vitro. Activity at the NMDA receptor was assessed using the calcium-sensitive probe Fura-2 (Molecular Probes-ThermoFisher, Eugene, OR, USA). After incubation with 6 μM Fura-2 acetoxymethyl ester (Fura-2 AM) for 30–45 min at 37 °C, a coverslip was transferred to a plastic holder that was inserted in a quartz cuvette for fluorescence measurements. Recordings of Fura-2 fluorescence were performed using a PerkinElmer LS55 luminiscence spectrometer, both at 340 and 380 nm excitation wavelengths, and at 510 nm of emission. The ratio of F340/F380 (R) is proportional to intracellular calcium. All the measurements were made at 37 °C and under mild stirring. Once the recording was started, NMDA (100 μM, in the presence of 10 μM glycine) was added to the cuvette. This produced a sustained increase in R, indicating the activation of the NMDA receptors and that the intracellular calcium concentration was high. After 400 s, this intracellular calcium increase was challenged with cumulative concentrations of the compounds under investigation, (from 1 *×* 10*^−^*^7^ M up to up to 3 *×* 10*^−^*^4^ M). If the compounds would act as antagonists at the NMDA receptor this would be detected as a decrease in the R value. Experiments were repeated 3 to 5 times, using different batches of cultures. Amantadine was used as a positive control. When a minimum of 50% of inhibition was reached, the IC_50_ value was calculated using non-linear regression with GraphPad Prism 5 (GraphPad Software Inc., San Diego, CA, USA).

## 4. Conclusions

Previous works have described that some indoloquinolizidines alkaloids act as NMDA receptor antagonists. However, the study of the indoloquinolizidine nucleous for the NMDA receptor antagonism has not been previously studied. The present work aimed at screening a small series of enantiopure indoloquinolizidines for their in vitro activity as NMDA receptor blockers. To achieve this objective, we have synthesized a series of enantiopure indolo[2,3-*a*]quinolizidines starting from (*S*)- or (*R*)-trypthophanol. The compounds were screened for NMDA receptor antagonistic activity using cerebellar granule neurons and one compound was identified to be 2.9 times more potent than the positive control amantadine and 2 times more active than the trypthophanol-derived lactam **4** previously identified by us as NMDA receptor antagonist. Besides its potential applicability in neurodegenerative diseases where NMDA receptor activity is exacerbated, compound **7a** is a promising starting point for the development of more potent derivatives targeting NMDA receptors.

## Figures and Tables

**Table 1:** IC_50_ values obtained for compounds **4**, **7a**, and amantadine.

Compound	NMDA (100 μM)
	IC_50_ μM ^a^
**4**	63.4 ± 9.0
**7a**	30.4 ± 2.5
**Amantadine**	88.5 ± 11.8

^a^ Data are expressed as the mean of three independent experiments.

## References

[1] Rouf A., Taneja S.C. (2014). Synthesis of single-enantiomer bioactive molecules: A brief overview. Chirality.

[2] Ager D.J., Prakash I., Schaad D.R. (1996). 1,2-amino alcohols and their heterocyclic derivatives as chiral auxiliaries in asymmetric synthesis. Chem. Rev..

[3] Pereira N.A.L., Sureda F.X., Turch M., Amat M., Bosch J., Santos M.M.M. (2013). Synthesis of phenylalaninol-derived oxazolopyrrolidone lactams and evaluation as nmda receptor antagonists. Monatsh. Chem..

[4] Pereira N.A.L., Sureda F.X., Esplugas R., Perez M., Amat M., Santos M.M.M. (2014). Tryptophanol-derived oxazolopiperidone lactams: Identification of a hit compound as nmda receptor antagonist. Bioorg. Med. Chem. Lett..

[5] Soares J., Raimundo L., Pereira N.A.L., dos Santos D.J.V.A., Perez M., Queiroz G., Leao M., Santos M.M.M., Saraiva L. (2015). A tryptophanol-derived oxazolopiperidone lactam is cytotoxic against tumors via inhibition of p53 interaction with murine double minute proteins. Pharmacol. Res..

[6] Soares J., Pereira N.A.L., Monteiro A., Leao M., Bessa C., dos Santos D.J.V.A., Rairnundo L., Queiroz G., Bisio A., Inga A. (2015). Oxazoloisoindolinones with in vitro antitumor activity selectively activate a p53-pathway through potential inhibition of the p53-mdm2 interaction. Eur. J. Pharm. Sci..

[7] Soares J., Raimundo L., Pereira N.A.L., Monteiro A., Gomes S., Bessa C., Pereira C., Queiroz G., Bisio A., Fernandes J. (2016). Reactivation of wild-type and mutant p53 by tryptophanol-derived oxazoloisoindolinone slmp53-1, a novel anticancer small-molecule. Oncotarget.

[8] Pereira N.A.L., Monteiro A., Machado M., Gut J., Molins E., Perry M.J., Dourado J., Moreira R., Rosenthal P.J., Prudencio M. (2015). Enantiopure indolizinoindolones with in vitro activity against blood- and liver-stage malaria parasites. Chem. Med. Chem..

[9] Paoletti P., Neyton J. (2007). Nmda receptor subunits: Function and pharmacology. Curr. Opin. Pharmacol..

[10] Morris R.G.M. (2013). Nmda receptors and memory encoding. Neuropharmacology.

[11] Santangelo R.M., Acker T.M., Zimmerman S.S., Katzman B.M., Strong K.L., Traynelis S.F., Liotta D.C. (2012). Novel nmda receptor modulators: An update. Expert Opin. Ther. Pat..

[12] Faulkner M.A. (2014). Safety overview of fda-approved medications for the treatment of the motor symptoms of parkinson’s disease. Expert Opin. Drug Saf..

[13] Hellweg R., Wirth Y., Janetzky W., Hartmann S. (2012). Efficacy of memantine in delaying clinical worsening in alzheimer’s disease (ad): Responder analyses of nine clinical trials with patients with moderate to severe ad. Int. J. Geriatr. Psychiatry.

[14] Lee J., Son D., Lee P., Kim S.Y., Kim H., Kim C.J., Lim E. (2003). Alkaloid fraction of uncaria rhynchophylla protects against n-methyl-d-aspartate-induced apoptosis in rat hippocampal slices. Neurosci. Lett..

[15] Shimada Y., Goto H., Itoh T., Sakakibara I., Kubo M., Sasaki H., Terasawa K. (1999). Evaluation of the protective effects of alkaloids isolated from the hooks and stems of uncaria sinensis on glutamate-induced neuronal death in cultured cerebellar granule cells from rats. J. Pharm. Pharmacol..

[16] Jung H.Y., Nam K.N., Woo B.-C., Kim K.-P., Kim S.-O., Lee E.H. (2013). Hirsutine, an indole alkaloid of uncaria rhynchophylla, inhibits inflammation-mediated neurotoxicity and microglial activation. Mol. Med. Rep..

[17] Imamura S., Tabuchi M., Kushida H., Nishi A., Kanno H., Yamaguchi T., Sekiguchi K., Ikarashi Y., Kase Y. (2011). The blood-brain barrier permeability of geissoschizine methyl ether in uncaria hook, a galenical constituent of the traditional japanese medicine yokukansan. Cell. Mol. Neurobiol..

[18] Ndagijimana A., Wang X., Pan G., Zhang F., Feng H., Olaleye O. (2013). A review on indole alkaloids isolated from uncaria rhynchophylla and their pharmacological studies. Fitoterapia.

[19] Allin S.M., Thomas C.I., Doyle K., Elsegood M.R.J. (2005). An asymmetric synthesis of both enantiomers of the indole alkaloid deplancheine. J. Org. Chem..

[20] Allin S.M., Thomas C.I., Allard J.E., Doyle K., Elsegood M.R.J. (2005). A highly stereoselective synthesis of the indolo 2,3-a quinolizine ring system and application to natural product synthesis. Eur. J. Org. Chem..

[21] Bassas O., Llor N., Santos M.M.M., Griera R., Molins E., Amat M., Bosch J. (2005). Biogenetically inspired enantioselective approach to indolo [2,3-*a*]- and benzo a quinolizidine alkaloids from a synthetic equivalent of secologanin. Org. Lett..

[22] Amat M., Santos M.M.M., Bassas O., Llor N., Escolano C., Gomez-Esque A., Molins E., Allin S.M., McKee V., Bosch J. (2007). Straightforward methodology for the enantioselective synthesis of benzo a - and indolo 2,3-a quinolizidines. J. Org. Chem..

[23] Amat M., Santos M.M.M., Gomez A.M., Jokic D., Molins E., Bosch J. (2007). Enantioselective spirocyclizations from tryptophanol-derived oxazolopiperidone lactams. Org. Lett..

[24] Amat M., Gomez Esque A., Escolano C., Santos M.M.M., Molins E., Bosch J. (2009). Enantioselective formal synthesis of (+)-dihydrocorynantheine and (−)-dihydrocorynantheol. J. Org. Chem..

[25] Perez M., Arioli F., Rigacci G., Santos M.M.M., Gomez-Esque A., Florindo P., Ramos C., Bosch J., Amat M. (2011). Stereocontrolled generation of benzo[α]- and indolo[2,3-α]quinolizidines from (*s*)-tryptophanol and (*s*)-(3,4-dimethoxyphenyl)alaninol-derived lactams. Eur. J. Org. Chem..

[26] Amat M., Ramos C., Perez M., Molins E., Florindo P., Santos M.M.M., Bosch J. (2013). Enantioselective formal synthesis of ent-rhynchophylline and ent-isorhynchophylline. Chem. Commun..

[27] Arioli F., Perez M., Are C., Estarellas C., Luque F.J., Bosch J., Amat M. (2015). Stereocontrolled annulations of indolo 2,3-a quinolizidine-derived lactams with a silylated nazarov reagent: Access to allo and epiallo yohimbine-type derivatives. Chem. Eur. J..

[28] Santos M.M.M., Carreiras M.C., Marco-Contelles J. (2011). Tryptophanol-derived oxazolopiperidone lactams: Valuable building blocks for the enantioselective synthesis of piperidine-containing alkaloids. Heterocyclic Targets in Advanced Organic Synthesis.

[29] Perez M., Espadinha M., Santos M.M.M. (2015). Indolo 2,3-a quinolizidines and derivatives: Bioactivity and asymmetric synthesis. Curr. Pharm. Des..

[30] Horrocks P., Fallon S., Denman L., Devine O., Duffy L.J., Harper A., Meredith E.-L., Hasenkamp S., Sidaway A., Monnery D. (2012). Synthesis and evaluation of a novel series of indoloisoquinolines as small molecule anti-malarial leads. Bioorg. Med. Chem. Lett..

[31] Amat M., Bassas O., Cantó M., Llor N., Santos M.M.M., Bosch J. (2005). Synthesis of 3-acetonyl- and 3-(2-oxoethyl)glutarates. Tetrahedron.

[32] Allin S.M., Gaskell S.N., Elsegood M.R.J., Martin W.P. (2007). A new asymmetric synthesis of the natural enantiomer of the indolizidino 8,7-b indole alkaloid (+)-harmicine. Tetrahedron Lett..

